# Free-living and laboratory gait characteristics in patients with multiple sclerosis

**DOI:** 10.1371/journal.pone.0196463

**Published:** 2018-05-01

**Authors:** Fabio A. Storm, K. P. S. Nair, Alison J. Clarke, Jill M. Van der Meulen, Claudia Mazzà

**Affiliations:** 1 Department of Mechanical Engineering, The University of Sheffield, Sheffield, United Kingdom; 2 INSIGNEO Institute for *in Silico* Medicine, The University of Sheffield, Sheffield, United Kingdom; 3 Department of Neurology, Sheffield Teaching Hospitals NHS Foundation Trust, Sheffield, United Kingdom; 4 The Gait Laboratory, Sheffield Teaching Hospitals NHS Foundation Trust, Sheffield, United Kingdom; University of Illinois at Urbana-Champaign, UNITED STATES

## Abstract

**Background:**

Wearable sensors offer the potential to bring new knowledge to inform interventions in patients affected by multiple sclerosis (MS) by thoroughly quantifying gait characteristics and gait deficits from prolonged daily living measurements. The aim of this study was to characterise gait in both laboratory and daily life conditions for a group of patients with moderate to severe ambulatory impairment due to MS. To this purpose, algorithms to detect and characterise gait from wearable inertial sensors data were also validated.

**Methods:**

Fourteen patients with MS were divided into two groups according to their disability level (EDSS 6.5–6.0 and EDSS 5.5–5.0, respectively). They performed both intermittent and continuous walking bouts (WBs) in a gait laboratory wearing waist and shank mounted inertial sensors. An algorithm (W-CWT) to estimate gait events and temporal parameters (mean and variability values) using data recorded from the waist mounted sensor (Dynaport, Mc Roberts) was tested against a reference algorithm (S-REF) based on the shank-worn sensors (OPAL, APDM). Subsequently, the accuracy of another algorithm (W-PAM) to detect and classify WBs was also tested. The validated algorithms were then used to quantify gait characteristics during short (sWB, 5–50 steps), intermediate (iWB, 51–100 steps) and long (lWB, >100 steps) daily living WBs and laboratory walking. Group means were compared using a two-way ANOVA.

**Results:**

W-CWT compared to S-REF showed good gait event accuracy (0.05–0.10 s absolute error) and was not influenced by disability level. It slightly overestimated stride time in intermittent walking (0.012 s) and overestimated highly variability of temporal parameters in both intermittent (17.5%–58.2%) and continuous walking (11.2%–76.7%). The accuracy of W-PAM was speed-dependent and decreased with increasing disability. The ANOVA analysis showed that patients walked at a slower pace in daily living than in the laboratory. In daily living gait, all mean temporal parameters decreased as the WB duration increased. In the sWB, the patients with a lower disability score showed, on average, lower values of the temporal parameters. Variability decreased as the WB duration increased.

**Conclusions:**

This study validated a method to quantify walking in real life in people with MS and showed how gait characteristics estimated from short walking bouts during daily living may be the most informative to quantify level of disability and effects of interventions in patients moderately affected by MS. The study provides a robust approach for the quantification of recognised clinically relevant outcomes and an innovative perspective in the study of real life walking.

## Introduction

Multiple sclerosis (MS) is a chronic autoimmune inflammatory disease of the central nervous system. The prevalence of mobility problems in people with MS is around 75%-90% [1,2], and gait impairment affects their general health status, quality of life and productivity [3]. As already shown in other patient populations [4], the quantification of gait deficits and changes in gait characteristics of people with MS is key to understand the progression of disability, and will also be a valuable tool to quantify the effect of different interventions.

Gait deficits in persons affected by MS have been traditionally identified using specialized equipment in confined gait laboratories, such as motion capture systems and pressure sensitive walkways [5]. These studies showed that patients with MS have slower walking speed, prolonged stride time and longer double support compared to controls [6]. Leveraging on the body of knowledge coming from laboratory-based observations and on the availability of wearable sensors able to monitor gait unobtrusively for long periods of time, the attention of the research is now moving toward the investigation of gait in real-life scenarios [7,8]. Thanks to these tools, it has been recently shown that gait variability in patients with MS is an early sign of disease progression [9,10] and is associated to higher risk of falls [11]. Validation studies have highlighted that, despite the fact that the accuracy of gait observations made using Physical Activity monitors (PAMs) in people with MS depend on walking speed and cadence [12–14], these tools can potentially provide an insight into interesting features observable in terms of variations in activity intensity, steps per day, or energy expenditure.

Whereas it is known that gait performance varies as a function of environment and walking bout length [15,16], what still is lacking in the literature concerning real life walking of patients with MS, is information about how changes in step-by-step characteristics might be associated with the length of the walking bouts from which they are extracted, and about how these relate back to data collected from a single observation in clinical or laboratory settings. As true also for other patient populations [17], the characterisation of gait parameters from daily living conditions in people with MS is certainly limited by the lack of algorithms specifically validated for this population. Algorithms based on foot or shank-mounted sensor data provide accurate estimation of gait events and temporal parameters [18–20]. Recently, a triaxial accelerometer placed on the shank was used to measure stride time, swing time and step time in 45 MS patients in controlled conditions [21]. However, these approaches have not yet been used to investigate walking bouts of patients with MS collected in free living conditions.

In the context of free living movement analysis, minimizing the instrumentation setup to a single device to maximise comfort and reduce alteration of the subject’s gait is desirable [22]. Waist-placement is often preferred for single sensor configurations, because the device is close to the centre of mass of the human body and hence thought to be better representing human motion [23]. Acceleration data collected at the pelvis level can also provide a wide range of clinically meaningful gait parameters, beyond the observation of spatio-temporal features [24].

The aim of this study is to quantify changes of gait characteristics of a group of patients with MS as observed both between laboratory and real life and as extracted from walking bouts of different lengths recorded in real life from MS patients with different disability levels. To this purpose, a method for gait event and temporal parameter estimation based on a single-sensor configuration located at the pelvis will also be validated. The results of this investigation will open the way to an innovative approach to the use of gait as a biomarker in multiple sclerosis and will shed some light about the link existing between the clinics and real life observation, which could be used for the definition of more tailored interventions.

## Materials and methods

### Participants

Recruitment and data collection took place at the Gait Laboratory, Northern General Hospital, Sheffield, UK. Written informed consent was obtained from the participants and ethical approval was obtained from NRES Committees—North of Scotland. Inclusion criteria for the participation in the study were: diagnosis of MS using McDonald’s criteria [25], three months since last relapse, and ability to independently walk for 10 meters. 14 participants were recruited in this study (seven men and seven women, age: 54.8 ± 11.0 years). The severity of MS was measured using Expanded Disability Status Scale (EDSS) [26].

### Equipment and protocol

The participants were outfitted with two types of wearable motion sensors. A PAM (MoveMonitor, Version 2.8.1, Mc Roberts, The Hague, The Netherlands) was positioned on the lower back of each participant by means of an elastic strap. The MoveMonitor is a triaxial accelerometer-based PAM, providing a classification of categories of physical activity (lying, sitting, standing, walking, shuffling) and step detection [27,28]. It collects acceleration signals at a sampling frequency of 100 Hz in a range of ± 2 g, and allows the extraction of raw data for further processing. Two inertial measurement units (OPAL, APDM Inc., Portland, OR, USA) were also strapped to each shank of the participant, just above the malleoli. These devices measured accelerations and angular velocities at a sampling frequency of 128 Hz (accelerometer range set at ± 6 g). The participants completed four intermittent walks along a predefined 15m straight walkway at their natural comfortable speed, measured by two light-gates. Then, they were asked to walk continuously for one minute in a 100 m^2^ empty room, without following any predefined path. This included walking in straight lines, turning, and walking in spirals. These two lab-based sessions were labelled “intermittent” and “continuous”, respectively. Then, the PAM was given to the participants for one week of continuous recording of their physical activity. They were asked to wear the device during the day and a valid day of wear time was defined as having ten or more hours of recorded data (Troiano et al., 2008).

### Data processing

The PAM raw triaxial acceleration signals were extracted and processed to obtain initial contact (IC) and final contact (FC) timings of each foot using an algorithm based on continuous wavelet transforms of the raw signals collected at the waist (W-CWT). The algorithm has been previously validated for healthy individuals both in laboratory [29] and free-living conditions [16]. For the W-CWT method, a first Gaussian continuous wavelet transformation is applied to the vertical acceleration signal, and the minima are identified as the IC timings. The resulting signal is then differentiated and the maxima are identified as the FC timings [29]. The W-CWT algorithm was selected for its reported robustness to changes in IMU attachments and gait speed, and for its reported high accuracy in comparison to other methods based on waist-worn sensors [30,31].

The raw acceleration and angular velocity signals collected by the shank sensors, were processed to detect the number of steps and the IC and FC timings using a method previously validated to investigate temporal gait parameters in a number of patient populations [20], which was here considered as reference method (S-REF). In this approach, the peak in the angular velocity signals in the sagittal plane during mid-swing is used to identify windows in the signal where no gait events can occur. When coupled with the alternate shank, these intervals allow the identification of search windows for IC and FC events. The IC is identified as the instant of minimum angular velocity in the sagittal plane between the beginning of the IC search window and the instant of maximum anterior-posterior acceleration. The FC is identified as the instant of minimum anterior-posterior acceleration in the FC search window [32].

For both W-CWT and S-REF average and variability (standard deviation, sd) values for stride time, step time, stance time, and swing time were calculated for each participant and for both laboratory-based walking protocols (intermittent and continuous). The S-REF algorithm was also used as a reference to evaluate the accuracy of the total number of steps and the percentage of total walking time that was classified correctly as locomotion by the PAM proprietary algorithm (W-PAM). The W-PAM method has been validated in several patient populations [33,34], and consists of five parts: gait period detection, transition detection, detection of lying and sitting, detection of shuffling, and detection of larger transitions [35].

After the seven days of monitoring, data from the PAM was downloaded and the raw acceleration signals corresponding to all walking bouts (WB), i.e. continuous times spent walking, longer than or equal to five steps were extracted using W-PAM. Data corresponding to WB shorter than five steps were discarded to avoid misinterpretation of intermittent stepping [36,37]. For each WB, IC and FC events were extracted using W-CWT and average and variability (sd) values were calculated for stride time, step time, stance time and swing time. These temporal gait parameters were selected because of their importance as indicators of gait pathology [38].

### Statistical analysis

The statistical analysis was conducted using SPSS (Version 21; SPSS Inc., Chicago, USA). Shapiro-Wilk tests of normality were initially performed on the measures. As the assumption of normality was not violated, parametric tests were used. For the purpose of validating W-CWT, data obtained in the laboratory from the two sessions was pooled together. The accuracy of the method was assessed computing the absolute error for IC and FC timings, as follows:

$$\left|E|=|p-p_{r}\right|$$

For the temporal parameters, in addition to |E|, also the percentage error was calculated as follows:

$$|E|\%=\frac{\left|p-p_{r}\right|}{p_{r}}*100$$

Where p_r_ is the reference value of the parameter *p*. Participants were also grouped according to the disability score: EDSS 6.5–6.0 and EDSS 5.5–5.0. Differences in gait event detection errors between the two groups were investigated with independent samples t-tests.

We designed this statistical section of the paper in order to test if the accuracy of the W-CWT method was influenced by the disability level. In this case, a two-way ANOVA could not be performed since the dependent variable (error |E|) was not repeated. Then, paired-samples t-tests were performed to test for differences between the parameters calculated using the W-CWT and the S-REF sensors.

For the accuracy assessment of W-PAM, laboratory data was used and the intermittent and the continuous walking trials were pooled together. Step detection accuracy of W-PAM (N_W-PAM_) was evaluated against the reference method (N_S-REF_) by calculating |E|%, as above.

For the daily living walking data, the WB were grouped according to the number of consecutive steps as follows: short (sWB, 5–50 consecutive steps), intermediate (iWB, 51–100 consecutive steps), long (lWB, +100 consecutive steps). Differences in gait parameters between these WBs as extracted from free-living, from intermittent and continuous laboratory trials and between patients with lower (EDSS = 5.0–5.5) and higher (EDSS = 6.0–6.5) disability scores were investigated with a two-way (walking bouts × disability group) ANOVA analysis, with Bonferroni correction for post-hoc tests. Differences with P-values lower than 0.05 were considered as statistically significant.

## Results

All participants took part in the laboratory visit. The EDSS score ranged between 5.0 (person able to walk without aid or rest for 200m) and 6.5 (person requiring two walking aids to walk 20m without resting).

Table 1 shows the gait event errors for W-CWT as obtained both for continuous and intermittent lab-based walking for the two groups. The FC showed a higher detection error than IC, while no differences in accuracy were found between intermittent and continuous walking. No statistically significant differences were found between the disability groups for any of the gait events or walking protocols.

**Table 1:** Initial and final contact absolute errors (|E|) for algorithm W-CWT summarized by group and walking protocol (mean ± SD).

|E| (s)	Protocol	EDSS 6.5–6.0	EDSS 5.5–5.0
Initial Contact	Intermittent	0.05 ± 0.03	0.06 ± 0.02
Initial Contact	Continuous	0.06 ± 0.02	0.07 ± 0.03
Final Contact	Intermittent	0.10 ± 0.04	0.10 ± 0.04
Final Contact	Continuous	0.10 ± 0.05	0.10 ± 0.03

Table 2 displays gait parameters calculated using W-CWT and S-REF, with the absolute and percentage errors for W-CWT. The statistical analysis showed that W-CWT slightly overestimated stride time in intermittent walking, and also overestimated swing time variability in both intermittent and continuous walking.

**Table 2:** Gait parameters calculated by the W-CWT and S-REF with absolute |E| and percentage errors |E|% calculated for W-CWT. Test statistic is provided for the statistically significant differences.

**MEAN VALUES**					
**Gait Parameter**	**Protocol**	**W-CWT**	**S-REF**	**|E|**_**W-CWT**_	**|E|%**_**W-CWT**_
Stride time (s)	Intermittent	1.53 ± 0.56* (t = 3.316)	1.54 ± 0.57	0.012 ± 0.012	0.7 ± 0.5
	Continuous	1.65 ± 0.56	1.65 ± 0.56	0.006 ± 0.005	0.4 ± 0.4
Step time (s)	Intermittent	0.76 ± 0.28* (t = 2.913)	0.77 ± 0.28	0.005 ± 0.005	0.7 ± 0.6
	Continuous	0.82 ± 0.28	0.82 ± 0.28	0.004 ± 0.004	0.4 ± 0.4
Stance time (s)	Intermittent	0.99 ± 0.40	1.02 ± 0.46	0.058 ± 0.049	5.1 ± 3.2
	Continuous	1.09 ± 0.45	1.12 ± 0.50	0.043 ± 0.043	3.2 ± 2.2
Swing time (s)	Intermittent	0.54 ± 0.16	0.52 ± 0.11	0.053 ± 0.040	9.6 ± 6.5
	Continuous	0.56 ± 0.12	0.53 ± 0.07	0.046 ± 0.044	8.1 ± 6.7
**VARIABILITY VALUES (sd)**					
**Gait Parameter**	**Protocol**	**W-CWT**	**S-REF**	**|E|**	**|E|%**
Stride time (s)	Intermittent	0.12 ± 0.09	0.12 ± 0.08	0.017 ± 0.013	17.5 ± 12.0
	Continuous	0.19 ± 0.12	0.18 ± 0.10	0.021 ± 0.025	11.2 ± 10.7
Step time (s)	Intermittent	0.11 ± 0.08	0.09 ± 0.06	0.032 ± 0.032	39.2 ± 34.3
	Continuous	0.17 ± 0.14	0.15 ± 0.10	0.039 ± 0.040	26.6 ± 23.1
Stance time (s)	Intermittent	0.15 ± 0.12	0.14 ± 0.10	0.033 ± 0.029	36.4 ± 37.8
	Continuous	0.19 ± 0.12	0.17 ± 0.10	0.041 ± 0.030	28.5 ± 22.6
Swing time (s)	Intermittent	0.11 ± 0.08* (t = 2.458)	0.08 ± 0.05	0.045 ± 0.038	58.2 ± 41.7
	Continuous	0.13 ± 0.08* (t = 3.602)	0.08 ± 0.05	0.056 ± 0.039	76.7 ± 58.1

*Statistically significantly different (p<0.05) from the reference S-REF method.

### Accuracy of the physical activity monitor in laboratory gait

The summary of the measures based on the level of disability (Table 3), showed that the number of steps detected (both N_S-REF_ and N_W-PAM_) decreased with increasing EDSS score. The accuracy of W-PAM and the walking speed of the participants also decreased considerably with increasing disability.

**Table 3:** Number of steps measured by the reference method (S-REF) and the PAM (W-PAM), percentage error |E|%, walking speed and classification of walking activity by the PAM for each disability group. Values are mean ± sd.

	EDSS 6.5–6.0	EDSS 5.5–5.0
**N**_**S-REF**_ **(steps)**	227 ± 90	304 ± 42
**N**_**W-PAM**_ **(steps)**	187 ± 114	252 ± 116
**Step detection |E|%**	27% ± 35%	19% ± 34%
**Walking speed (m/s)**	0.7 ± 0.3	1.0 ± 0.4
**% Locomotion**	84 ± 27	96 ± 9

**Fig 1:**
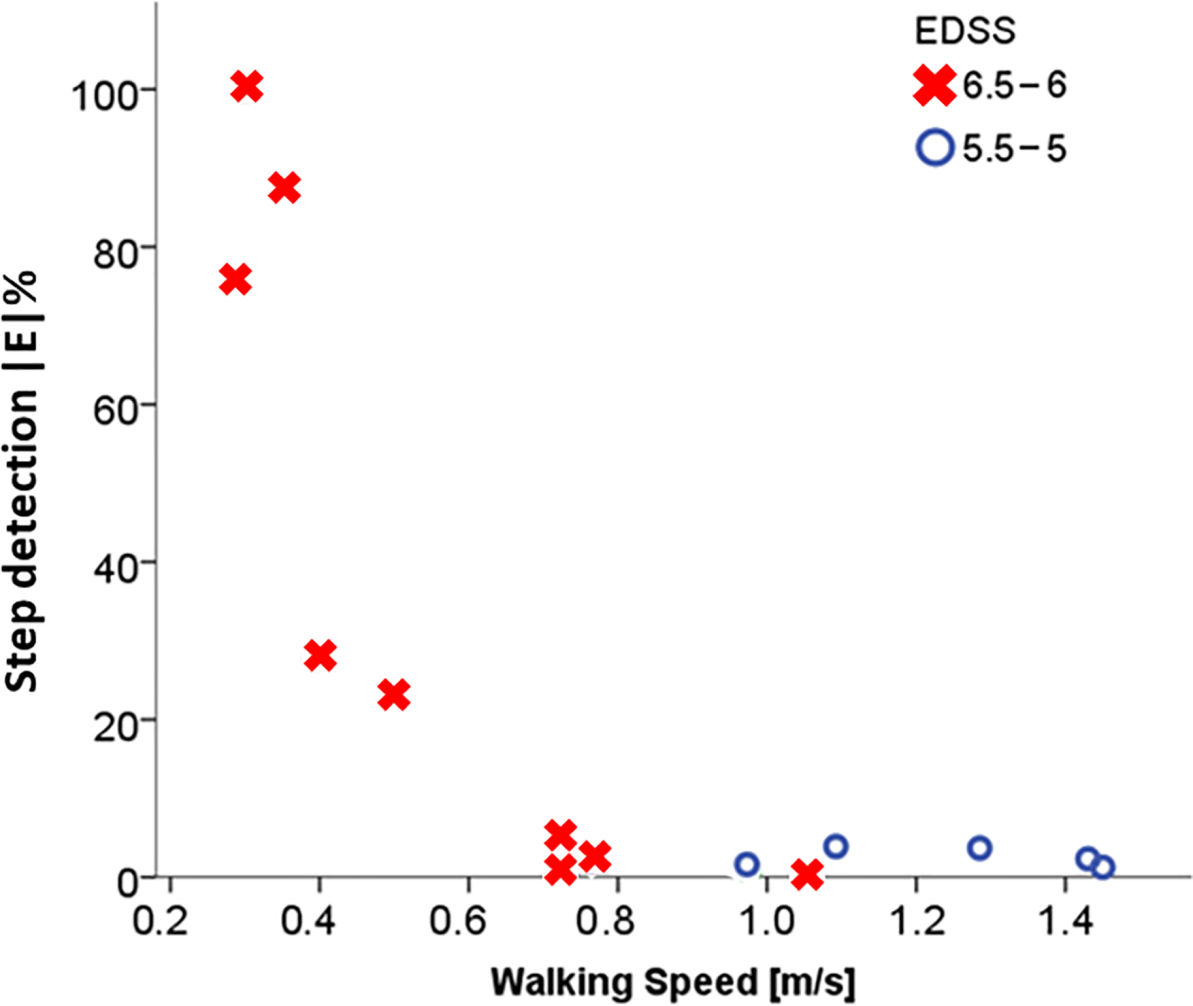
Relationship between mean percentage error for step detection (|E|%) of the algorithm W-PAM and walking speed according to disability group (EDSS score). All the participants with disability levels of 5.0 or 5.5 show very low errors and walking speeds above 0.8 m/s. For participants with disability levels of 6.0 or 6.5 the errors increase, particularly at the slowest walking speeds.

The overall mean percentage error for the number of steps detected by W-PAM was above 20% for five participants, all having an EDSS score of 6.5, and all walking below 0.6 m/s walking speed. For three of them, the |E|% for step detection was above 60%. For all the participants with an EDSS score of 5.0 the mean percentage error resulted below 4% and all of them walked above 1.0 m/s walking speed (Fig 1).

### Comparison of gait characteristics of free living and laboratory walking bouts

Data from three participants were excluded from the analysis of daily living gait because of the very high (>60%) step detection error measured for W-PAM in their laboratory gait sessions. The amount of sWB, iWB and lWB as a percentage of the total number of free living WBs were 58%, 22% and 21%, respectively. Table 4 provides the results of the ANOVA analysis for each investigated parameter. In addition, all temporal parameter values calculated for daily living gait (sWB, iWB, lWB) and laboratory gait (intermittent and continuous) are provided in the supporting information (S1 Table).

**Table 4:** Results of the two-way (walking bouts × disability group) ANOVA analysis performed to investigate differences in gait temporal parameters obtained in daily living and laboratory gait and between patients with lower (EDSS = 5.0–5.5) and higher (EDSS = 6.0–6.5) disability. The p-values are highlighted in bold when they indicate a significant effect (p<0.05). Significant differences in groups after post-hoc analysis are provided for the interaction effect.

		Interaction Effect	Main Effects	
		WBs x Disability group	Disability group	WBs
	Parameter	p-value	p-value	p-value
**MEAN VALUES**	Stride Time	0.034*	0.376	**<0.001**
	Step Time	0.032*	0.383	**<0.001**
	Stance Time	0.049*	0.166	**<0.001**
	Swing Time	0.033*	0.858	**<0.001**
**VARIABILITY**	Stride Time	0.631	0.784	**<0.001**
	Step Time	0.132	0.346	**<0.001**
	Stance Time	0.561	0.286	**<0.001**
	Swing Time	0.109	0.915	**<0.001**

*Significant Post-hoc test: sWB EDSS 6.5–6.0 > sWB EDSS 5.5–5.0, p<0.05.

**Fig 2:**
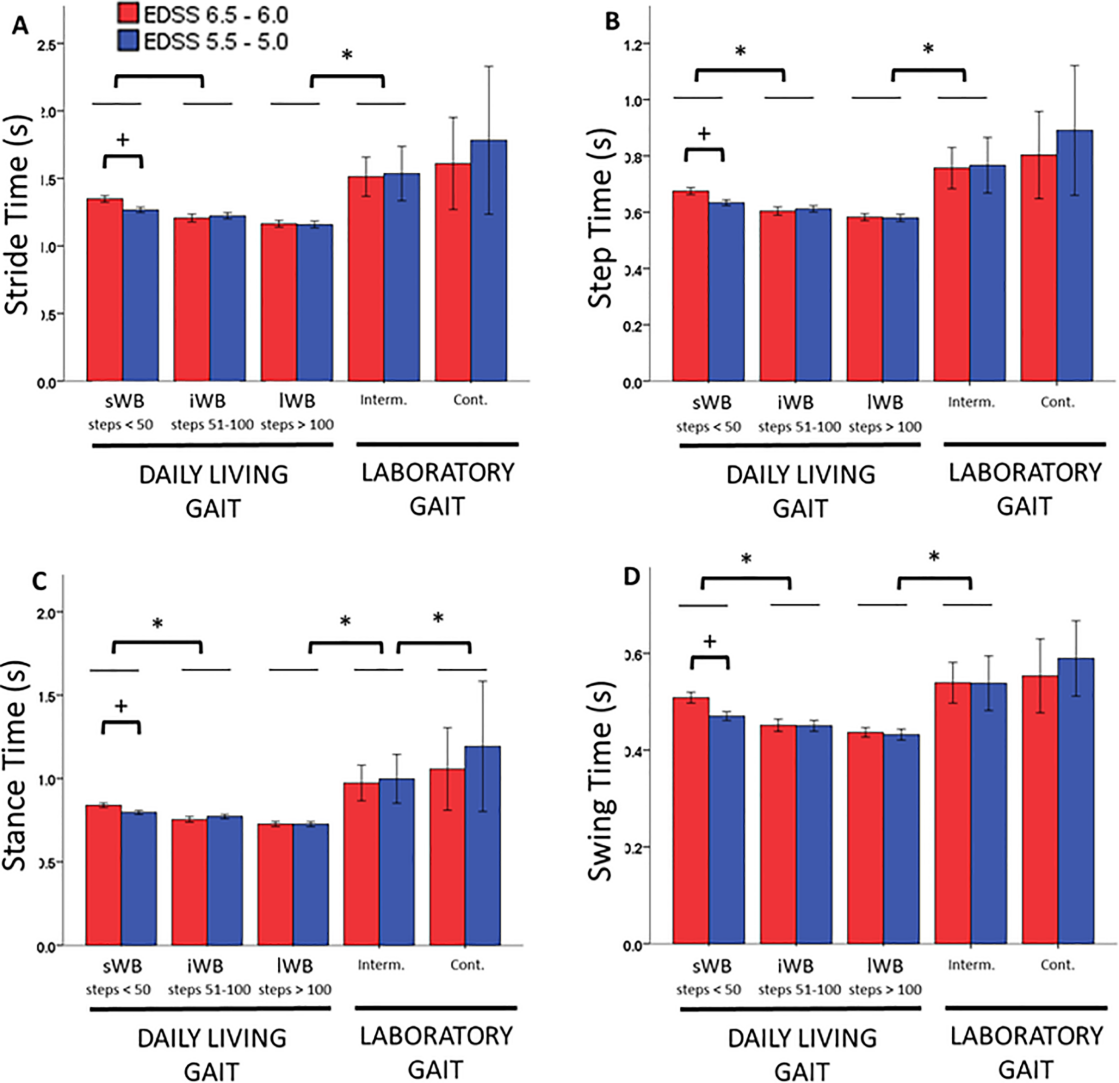
Temporal parameters. The figure depicts the average values of stride time (A), step time (B), stance time (C) and swing time (D) for the disability groups with EDSS 6.5–6.0 (red) and EDSS 5.5–5.0 (blue) for the daily life walking bouts (sWB, iWB and lWB) and the laboratory gait (intermittent and continuous). Values are mean and 95% CI. * Significant difference between WB types, p < 0.05. ^+^ Significant difference between disability groups, p<0.05.

There was a significant main effect of the type of walking bout (p<0.001) and a significant interaction effect between type of walking bout and disability group (p<0.05) for all the investigated mean parameters, and all the differences were higher than the errors expected for the relevant parameter. Daily living gait was characterized by higher pace than laboratory gait, regardless of the type or duration of the walking bouts. For daily living gait, mean values statistically significantly decreased as the WB duration increased, while for laboratory gait, the mean temporal parameters were higher during intermittent than during continuous gait, but the differences were not statistically significant. Post-hoc analysis for the significant interaction effect also revealed that the mean values were statistically significantly smaller in the group with less disability (EDSS 5.5–5.0) during the sWB (< 50 consecutive steps). Fig 2 shows the values obtained for the mean temporal parameters.

**Fig 3:**
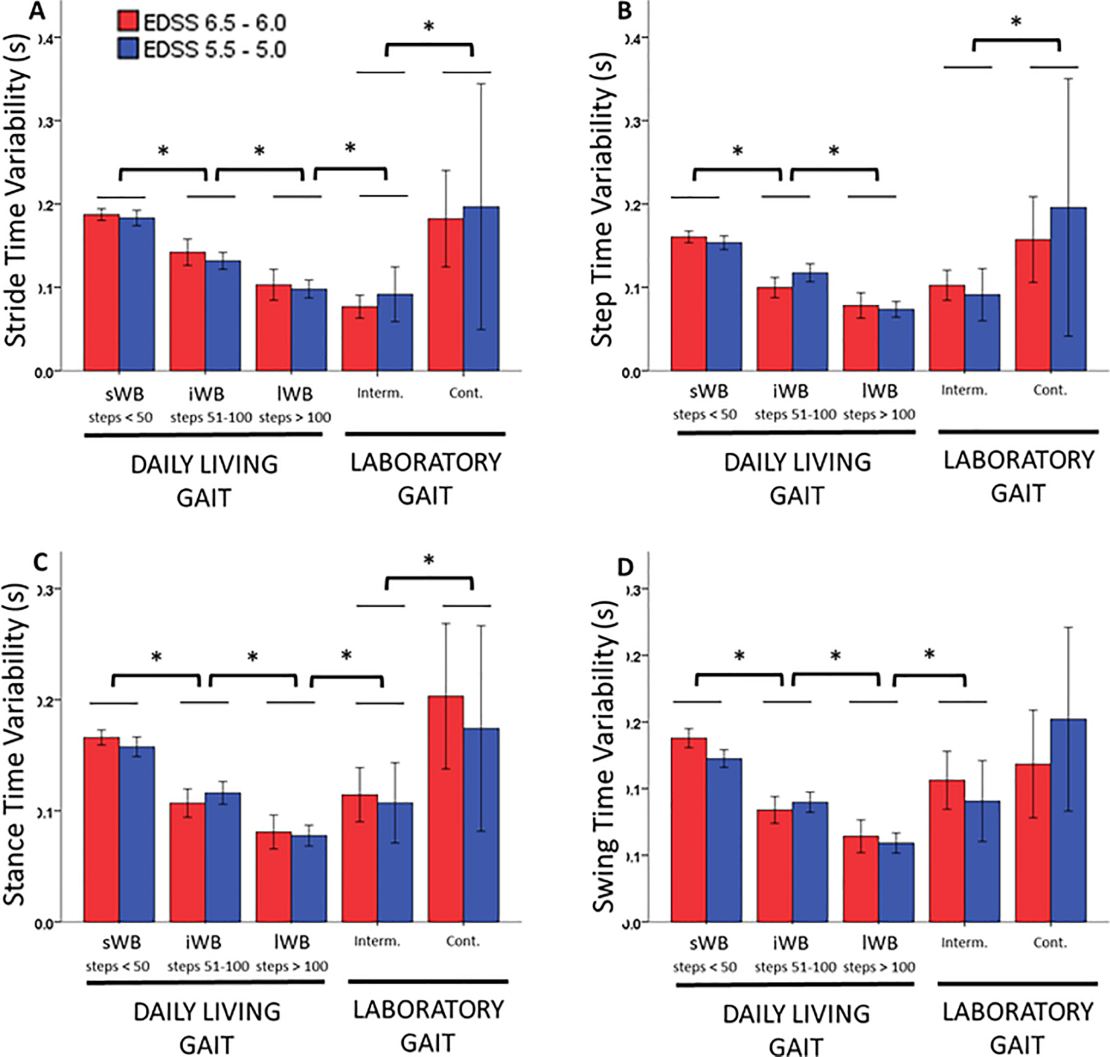
Variability of temporal parameters. The figure depicts the variability values of stride time (A), step time (B), stance time (C) and swing time (D) for the disability groups with EDSS 6.5–6.0 (red) and EDSS 5.5–5.0 (blue) for the daily life walking bouts (sWB, iWB and lWB) and the laboratory gait (intermittent and continuous). Values are mean and 95% CI. * Significant difference between WB types, p < 0.05.

For the temporal variability parameters, the ANOVA analysis showed a statistically significant main effect of WB type (p<0.001). For daily living gait, variability statistically significantly decreased as the WB duration increased, while for laboratory gait, continuous WBs were characterized by statistically significantly higher variability than intermittent WBs. When comparing daily living and laboratory gait, variability of the sWBs in daily living was not statistically different from continuous WBs performed in the laboratory, while the variability of the lWB in daily life was not statistically different from the intermittent laboratory gait. Fig 3 shows the values obtained for the variability of the temporal parameters.

## Discussion

The objective of this study was to gather an insight into differences occurring between gait characteristics from daily living and laboratory walking bouts for a group of patients with moderate to severe ambulatory impairment due to MS. To this purpose, the accuracy in gait events and gait temporal parameter estimation of an algorithm based on a single waist-worn sensor (W-CWT), originally proposed by McCamley et al. (2012), was also quantified.

The accuracy of W-CWT was in the range 0.05–0.07s for the initial contact, IC, and 0.10s for the final contact, FC. The mean errors were comparable to those recently provided in a validation study in healthy participants [16]. The higher error for FC is likely due to the smoother movement occurring during FC making the gait event less apparent to detect [20]. No differences in accuracy for IC and FC were found between intermittent and continuous laboratory walking bouts. These findings suggest that this method of estimation of daily living walking bouts could be used in real life, where walking conditions are varied and changes in walking direction are frequent. Finally, there were no statistically significant differences in accuracy between patients with higher or lower disability levels, as established according to the EDSS score. A recently published study showed similar findings for gait parameters measured using a method based on shank-worn sensors [21]. The present work shows that this can be achieved also with a single waist-worn sensor. This is a promising finding considering the gait impairment in our group of patients, which suggests that analysis of free-living gait could be accurately performed in patients with MS with EDSS score of 5.0 to 6.5.

When looking at the temporal parameter estimation, the W-CWT method overestimated stride time and step time by less than 1% during intermittent walking, while stance time was on average underestimated by up to 5% and swing time was overestimated by 9%. The latter value is comparable to findings of a previous study assessing gait temporal parameter in a MS population using shank-worn sensors, in which an error of 6% on average was reported [21]. Inaccuracies in the estimation of mean stance and swing time have consistently been reported as higher compared to stride and step times for methods based on waist-worn sensors [39] and shank-worn sensors [18,20]. A possible source of inaccuracy in the W-CWT method might be due to inherent weakness of methods based on wavelet transforms to rely on the periodicity of walking [40].

In terms of variability of gait temporal parameters, W-CWT overestimated the values compared to the reference method (S-REF). The most accurate value of variability was obtained for stride time, with errors of 17.5% and 11.2% for intermittent and continuous walking, respectively. Errors were higher for step time variability and stance time variability (up to 39.2 and 36.4%). The least accurate was swing time variability, with an overestimation of up to 76.6%. Previous studies have already shown that small errors in gait event detection affect variability measures more than mean values. A study comparing step time variability measured with a pressure mat and a handheld video camera in patients with early stage Parkinson’s Disease and healthy controls showed discrepancies of 55% and 30%, respectively [41]. Our study confirms that variability measures are highly sensitive to incorrect gait event identification, and that further improvements of the existing algorithms are needed if they want to be investigated from real-life data collected form patient populations.

Before the detection of specific steps, the analysis of daily living walking bouts required the latter to be identified. The accuracy of the PAM proprietary algorithm (W-PAM) in WB detection was hence also assessed. This analysis showed that the error in step detection was higher at the lower paces, which confirms previous findings about the difficulty of detecting steps at slow walking speeds in patients affected by MS [13,42]. The highest errors were found for participants with EDSS score of 6.5. This is in line with a recent study testing the ActivPAL3 device in a group of people moderately affected by MS (EDSS 4–6.5). For patients with EDSS score of 6.5, the tested device underestimated steps and walking duration by up to 60% and 47%, respectively [43]. This finding suggests that at moderate disability levels a quantification of the walking speed of the participant may be useful as inclusion criterion for studies investigating stepping and walking bout characteristics in daily life.

Once the confidence level for the adopted algorithms had been established, data from free living gait were investigated and compared to laboratory walking, to establish whether these would, as expected, provide complementary information. In free-living conditions both groups performed a larger number of short WB rather than longer WB, reflecting habitual behaviours as reported in previous research for other populations [44,45]. Mean temporal parameters decreased with increasing walking bout duration. For example, stride time decreased from 1.35s (sWB) to 1.21s (iWB) to 1.16s (lWB). This finding confirm previous research of free-living walking behaviour in clinical populations, which concluded that the average gait cycle duration of WBs decreased as the WB duration increased [44]. This supports the hypothesis that stride time increases when walking shorter distances [46]. Differences in mean temporal parameters between disability groups were significant only for the short bouts (<50 steps), but not for intermediate or long walking bouts. This suggests that for MS patients these walking bouts (<50 steps) may be the most informative to determine the level of disability. A previous research in PD patients investigating gait parameters in daily living [45] showed that temporal characteristics of WB of similar duration (between 30s and 60s) were sensitive to pathology (i.e. values were different between controls and the patients’ group). Further studies are needed to confirm this finding on larger sample sizes, reflecting a wider range of EDSS scores, and breaking down walking bouts into smaller groups.

Laboratory gait was characterized by longer stride times (1.51 s and 1.61 s for intermittent and continuous walking, respectively) and step times (0.76 s and 0.80 s) with respect to daily living WBs (maximum stride time of 1.16 s and step time of 0.58 s, both measured during lWB). A possible explanation for this is that people in supervised tests tend to decrease cadence (i.e. increase stride/step time) while increasing step length and velocity [47].

Variability of temporal parameters was generally higher in the free living environment than during intermittent walking in the laboratory, confirming previous findings in older adults [48–50]. However, the variability of continuous walking bouts in the laboratory was comparable to the short (<50 steps) bouts performed in daily living. This suggests that intermittent walking may relate to gait performance, while continuous walking better mimics everyday gait capacity. Differences in variability between disability groups were negligible considering the error range measured for the relevant parameters. Contrarily to previous findings in clinical populations [45], in our cohort between-group differences in gait characteristics were not exaggerated in daily living bouts. However, differences in disability score between our two groups were small and bigger sample sizes are required to verify this hypothesis.

## Conclusion

This study provided a new insight into the investigation of free living gait in patients with MS, highlighting how these patients are more inclined to walk for shorter than longer bouts, with their average cadence increasing when walking for a longer time. In addition, the patients with a more severe disability level showed higher values for stride, step, stance and swing times during the short bouts (number of consecutive steps <50), but not during intermediate or long walking bouts, suggesting that the latter may be the most informative to determine the level of disability. The investigated daily living gait biomarkers, based on the quantification of recognised clinically relevant outcomes, are excellent candidate to support the definition of more tailored interventions in patients moderately affected by MS.

## Supporting information

S1 Table Gait parameters measured during the 7 days of physical activity monitoring, divided by disability group and WB duration (in consecutive steps). *Statistically significantly different (p<0.05) from the EDSS 6.5–6.0 group. Mean difference between disability groups and 95% confidence intervals (CI) are also reported.
(DOCX)
