# Supplementary material for: Free-living and laboratory gait characteristics in patients with multiple sclerosis
Source: PLoS One. 2018 May 1;13(5):e0196463. doi: 10.1371/journal.pone.0196463 (PMC5929566; doi:10.1371/journal.pone.0196463)
Supplement: S1 Table — *Statistically significantly different (p<0.05) from the EDSS 6.5–6.0 group. Mean difference between disability groups and 95% confidence intervals (CI) are also reported. (DOCX) [file pone.0196463.s001.docx]

**S1 Table.** **Gait parameters measured during the 7 days of physical activity monitoring, divided by disability group and WB duration (in consecutive steps).** *Statistically significantly different (p<0.05) from the EDSS 6.5 - 6.0 group. Mean difference between disability groups and 95% confidence intervals (CI) are also reported.

| **MEAN VALUES** |  |  |  |  |  |
| --- | --- | --- | --- | --- | --- |
| **Gait Parameter** | **WB type** | **EDSS 6.5 - 6.0** | **EDSS 5.5 - 5.0** | **Mean diff** | **95% CI** |
| Stride Time (s) | sWB (steps < 50) | 1.35 ± 0.22 | 1.27 ± 0.15 * | 0.08 | 0.04 - 0.13 |
|  | iWB (steps 51-100) | 1.21 ± 0.12 | 1.22 ± 0.13 | -0.02 | -0.10 - 0.06 |
|  | lWB (steps > 100) | 1.16 ± 0.09 | 1.16 ± 0.15 | 0.01 | -0.08 - 0.09 |
|  | Lab Intermittent | 1.51 ± 0.60 | 1.54 ± 0.58 | -0.02 | -0.13 - 0.08 |
|  | Lab Continuous | 1.61 ± 0.56 | 1.78 ± 0.66 | -0.17 | -0.40 - 0.06 |
| Step Time (s) | sWB (steps < 50) | 0.67 ± 0.11 | 0.63 ± 0.07 * | 0.04 | 0.02 - 0.06 |
|  | iWB (steps 51-100) | 0.60 ± 0.06 | 0.61 ± 0.06 | -0.01 | -0.05 - 0.03 |
|  | lWB (steps > 100) | 0.58 ± 0.05 | 0.58 ± 0.07 | 0.00 | -0.04 - 0.04 |
|  | Lab Intermittent | 0.76 ± 030 | 0.77 ± 0.29 | -0.01 | -0.06 - 0.04 |
|  | Lab Continuous | 0.80 ± 0.28 | 0.89 ± 0.33 | -0.09 | -0.20 - 0.03 |
| Stance Time (s) | sWB (steps < 50) | 0.84 ± 0.14 | 0.80 ± 0.09 * | 0.04 | 0.01 - 0.07 |
|  | iWB (steps 51-100) | 0.76 ± 0.07 | 0.77 ± 0.07 | -0.02 | -0.07 - 0.04 |
|  | lWB (steps > 100) | 0.73 ± 0.06 | 0.73 ± 0.08 | 0.00 | -0.06 - 0.06 |
|  | Lab Intermittent | 0.97 ± 0.44 | 0.10 ± 0.43 | -0.02 | -0.1 - 0.05 |
|  | Lab Continuous | 1.06 ± 0.45 | 1.19 ± 0.55 | -0.14 | -0.29 - 0.02 |
| Swing Time (s) | sWB (steps < 50) | 0.50 ± 0.10 | 0.47 ± 0.07 * | 0.04 | 0.02 - 0.05 |
|  | iWB (steps 51-100) | 0.45 ± 0.05 | 0.45 ± 0.06 | 0.00 | -0.03 - 0.03 |
|  | lWB (steps > 100) | 0.44 ± 0.04 | 0.43 ± 0.06 | 0.01 | -0.02 - 0.03 |
|  | Lab Intermittent | 0.54 ± 0.17 | 0.54 ± 0.16 | 0.00 | -0.04 - 0.04 |
|  | Lab Continuous | 0.55 ± 0.14 | 0.59 ± 0.11 | -0.04 | -0.12 - 0.05 |

| **VARIABILITY** |  |  |  |  |  |
| --- | --- | --- | --- | --- | --- |
| **Gait Parameter** | **WB type** | **EDSS 6.5 - 6.0** | **EDSS 5.5 - 5.0** | **Mean diff** | **95% CI** |
| Stride Time (s) | sWB (steps < 50) | 0.19 ± 0.06 | 0.18 ± 0.07 | 0.01 | -0.01 - 0.02 |
|  | iWB (steps 51-100) | 0.14 ± 0.06 | 0.13 ± 0.06 | -0.02 | -0.04 – 0.00 |
|  | lWB (steps > 100) | 0.10 ± 0.07 | 0.10 ± 0.06 | 0.01 | -0.02 - 0.03 |
|  | Lab Intermittent | 0.08 ± 0.06 | 0.09 ± 0.09 | 0.01 | -0.02 - 0.04 |
|  | Lab Continuous | 0.18 ± 0.10 | 0.20 ± 0.18 | -0.04 | -0.10 - 0.02 |
| Step Time (s) | sWB (steps < 50) | 0.16 ± 0.06 | 0.15 ± 0.06 | 0.00 | -0.01 - 0.02 |
|  | iWB (steps 51-100) | 0.10 ± 0.05 | 0.12 ± 0.06 | 0.01 | -0.01 - 0.03 |
|  | lWB (steps > 100) | 0.08 ± 0.06 | 0.07 ± 0.05 | 0.01 | -0.02 - 0.03 |
|  | Lab Intermittent | 0.10 ± 0.07 | 0.09 ± 0.09 | -0.02 | -0.04 - 0.01 |
|  | Lab Continuous | 0.16 ± 0.09 | 0.20 ± 0.22 | -0.01 | -0.07 - 0.04 |
| Stance Time (s) | sWB (steps < 50) | 0.17 ± 0.06 | 0.16 ± 0.06 | 0.01 | 0.00 - 0.02 |
|  | iWB (steps 51-100) | 0.11 ± 0.05 | 0.12 ± 0.06 | -0.01 | -0.03 - 0.01 |
|  | lWB (steps > 100) | 0.08 ± 0.06 | 0.08 ± 0.05 | 0.00 | -0.02 - 0.02 |
|  | Lab Intermittent | 0.11 ± 0.10 | 0.11 ± 0.11 | 0.01 | -0.02 - 0.03 |
|  | Lab Continuous | 0.20 ± 0.12 | 0.17 ± 0.13 | 0.03 | -0.03 - 0.09 |
| Swing Time (s) | sWB (steps < 50) | 0.14 ± 0.06 | 0.12 ± 0.05 * | 0.02 | 0.01 - 0.03 |
|  | iWB (steps 51-100) | 0.08 ± 0.04 | 0.09 ± 0.04 | -0.01 | -0.02 - 0.01 |
|  | lWB (steps > 100) | 0.06 ± 0.05 | 0.06 ± 0.04 | 0.01 | -0.01 - 0.02 |
|  | Lab Intermittent | 0.11 ± 0.09 | 0.09 ± 0.09 | 0.02 | -0.01 - 0.04 |
|  | Lab Continuous | 0.12 ± 0.07 | 0.15 ± 0.10 | -0.03 | -0.08 - 0.02 |
